# Pharmacological activities and mechanisms of action of hypophyllanthin: A review

**DOI:** 10.3389/fphar.2022.1070557

**Published:** 2023-01-09

**Authors:** Wan Azmira Wan Saidin, Ibrahim Jantan, Siti Mariam Abdul Wahab, Juriyati Jalil, Mazlina Mohd Said, Syaratul Dalina Yusoff, Khairana Husain

**Affiliations:** ^1^ Centre for Drug and Herbal Development, Faculty of Pharmacy, Universiti Kebangsaan Malaysia, Kuala Lumpur, Malaysia; ^2^ Institute of Systems Biology, Universiti Kebangsaan Malaysia, Selangor, Malaysia

**Keywords:** hypophyllanthin, *Phyllanthus* sp., pharmacological activities, mechanistic studies, immunomodulating activities

## Abstract

Hypophyllanthin is a major lignan present in various *Phyllanthus* species and has been used as one of the bioactive chemical markers for quality control purposes as it contributes to their diverse pharmacological activities. The objective of this study is to compile up-to-date data on the pharmacological actions and mechanisms of hypophyllanthin. This review also includes the extracts of *Phyllanthus* species whose pharmacological actions have been partially attributed to hypophyllanthin. The scientific findings on the compound are critically analyzed and its potential as a lead molecule for the discovery of drug candidates for the development of therapeutics to treat diverse diseases is highlighted. Data collection was mainly through the exploration of Ovid-MEDLINE, Scopus, Science Direct, and Elsevier databases. Studies conducted *in vitro* and *in vivo* showed that hypophyllanthin had potent immunomodulating properties as well as a variety of other pharmacological properties, including anti-inflammatory, hepatoprotective, anti-tumor, anti-allergic, anti-hypertensive, and phytoestrogenic properties. Several mechanisms of action on the effects of hypophyllanthin on the immune system, in cancer and other disease states, were presented to provide some insights into its pharmacological effects. Before being submitted to clinical investigations, additional animal studies utilising different animal models are necessary to analyse its bioavailability, pharmacokinetics, and pharmacodynamic properties, as well as its toxicity, to determine its efficacy and safety. Understanding its potential as a lead molecule for the discovery of therapeutic candidates, particularly for the development of therapies for inflammatory and immune-related disorders, requires an understanding of its pharmacological activities and mechanisms of action. An insight into its pharmacological activities and mechanisms of action will provide an understanding of its potential as a lead compound for the discovery of drug candidates, especially for the development of therapies for inflammatory and immune related diseases.

## 1 Introduction

**FIGURE 1:**
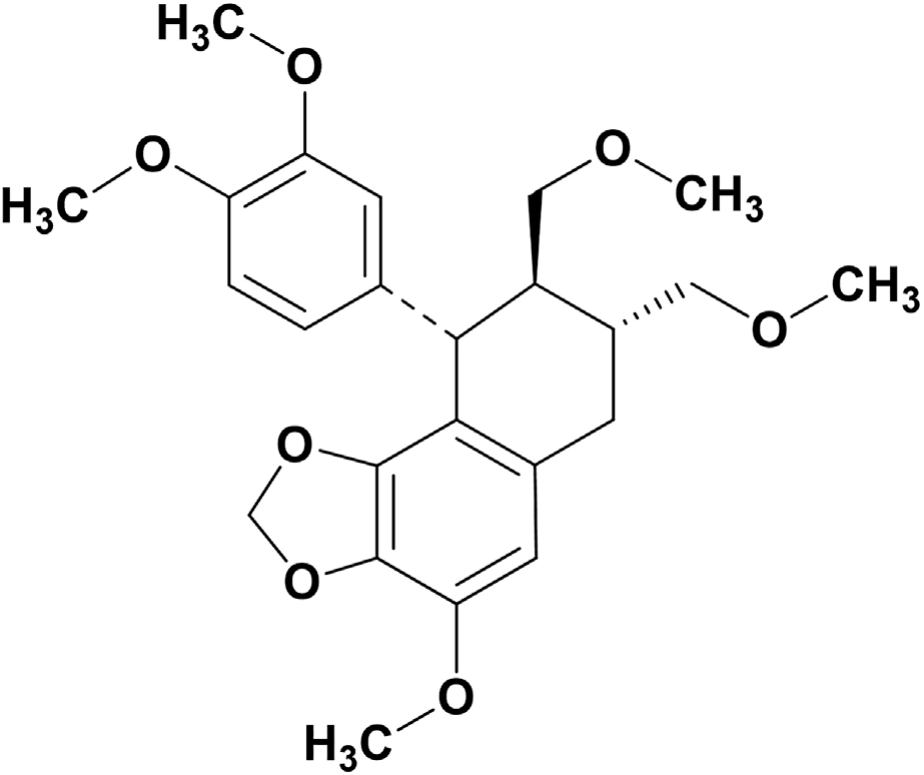
Structure of hypophyllanthin.

**FIGURE 2:**
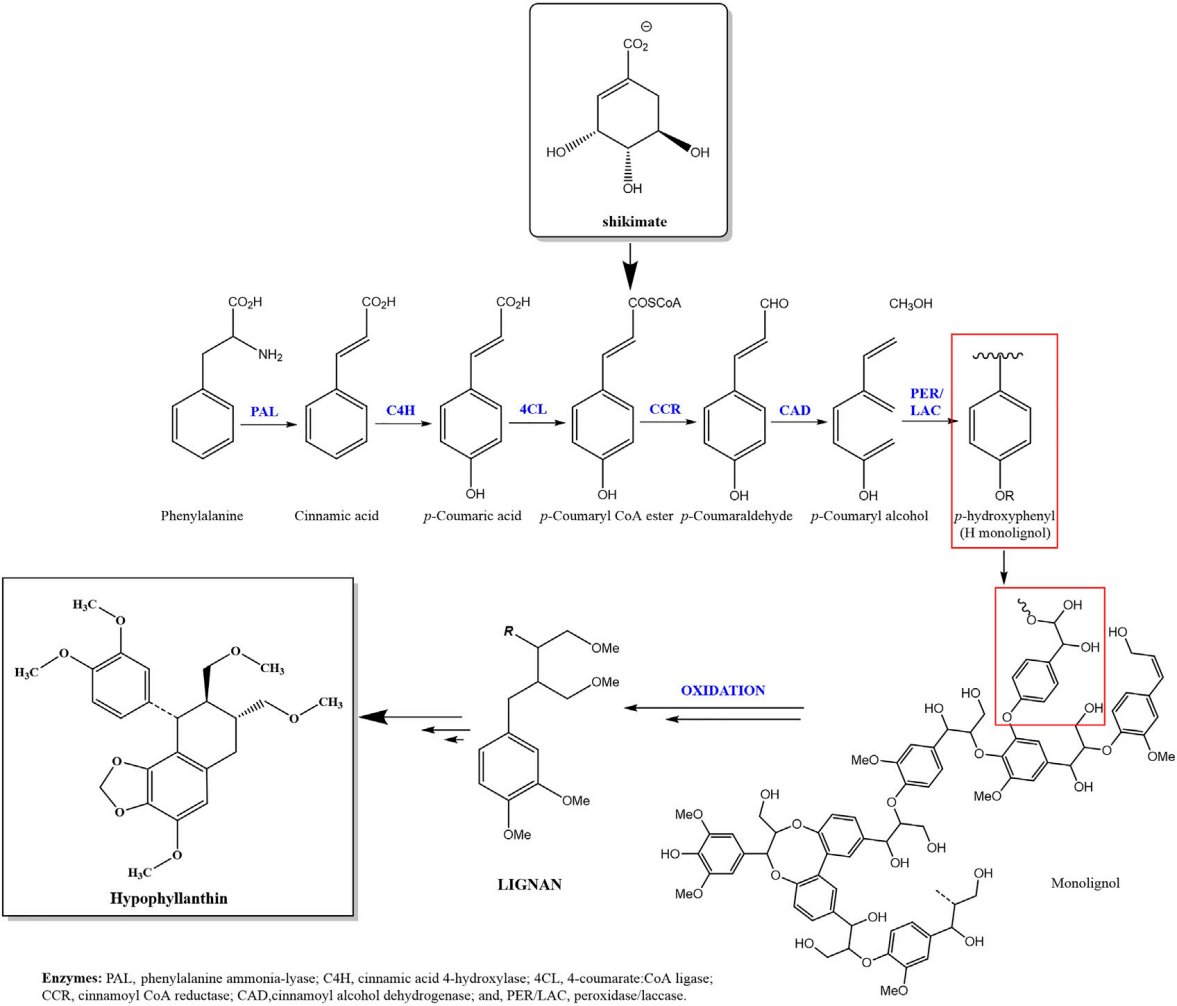
Proposed biosynthetic pathway for hypophyllanthin.

Hypophyllanthin is a naturally occurring lignan found in *Phyllanthus* species such as *P. amarus* Schum. and Thonn.*, P. niruri* L.*, P. urinaria* L*., P. debilis* J.G.Klein ex Willd*., P. virgatus* G. Forst., *P. fraternus* G.L.Webster*, P. maderaspatensis* L. and *P. simplex* var. *Simplex* Retz. Hypophyllanthin is chemically known as (7R,8R, 9S)-9-(3,4-dimethoxyphenyl)-4-methoxy-7,8-bis(methoxymethyl)-6,7,8,9-tetrahydrobenzo[g][1,3]benzodioxole with a molecular formula of C_24_H_30_O_7_ and molecular weight of 430.5 g/mol. Figure 1 shows the chemical structure of hypophyllanthin. The biosynthesis of hypophyllanthin involves the dimerization of cinnamic acid *via* the shikimate and phenylpropanoid pathways (Mendoza and Silva, 2018). Figure 2 shows the proposed biosynthesis pathway of hypophyllanthin (Lewis and Davin, 1999; Biała and Jasiński, 2018). The production of synthetic hypophyllanthin was described by Ganeshpure et al. (1981) as the reaction of 3-(3-methoxy-4,5-methylenedioxybenzyl)butyrolactone with bromine in acetic acid to produce 2-bromo-3-methoxy-4,5-methylenedioxybenzylbutyrolactone, which was subsequently reduced and methylated to produce hypophyllanthin.

*Phyllanthus* species have been utilised as a traditional medicine to cure a variety of ailments*.* For instance, in Ayurvedic medicine, *P. emblica* L. has been used extensively to heal different types of minor ailments like a fever as well as more serious illnesses such as diabetes (Nain et al., 2012) and skin problems (Adil et al., 2010). *P. niruri* and *P. reticulatus* Poir. are primarily used in traditional Chinese medicine to treat inflammation, urinary tract infection, rheumatoid arthritis, and ophthalmic diseases (Nain et al., 2012). It was also reported that the Brazilian and most of the South American communities have been using *P. niruri, P. tenellus* Roxb., *P. urinaria, P. caroliniensis* Walter*, P. corcovadensis* Müll.Arg., *P. stipulatus* (Raf.) G.L.Webster*, P. amarus, P. fraternus* G.L.Webster*,* and *P. sellowianus* (Klotzsch) Müll.Arg. to treat hepatitis B, diabetes, and infection in the intestines as well as the urinary bladder (Calixto et al., 1998). *P. amarus* is commonly used in Asia, particularly in Ayurvedic medicine, to treat genitourinary system, and spleen diseases, menorrhagia, stomach, liver, kidney, gonorrhoea, fevers, diarrhoea, ulcers, and wounds, whereas *P. niruri* is commonly used to treat inflammation, fever, malaria, lithiasis, and gonorrhoea. In Thailand, *P. urinaria*, *P. amarus*, and *P. virgatus* are used to treat gonorrhoea, diabetes, jaundice, and hepatic disorders, while *P. niruri*, *P. acidus*, and *P. reticulatus* are used to treat hypertension, malarial fever, constipation, and skin and urinary problems, respectively (Jantan et al., 2019). *P. niruri*, *P. simplex*, and *P. reticulatus* are used in China to treat inflammation, urinary tract infections, rheumatism, and ophthalmic disorders (Nain et al., 2012).

Thus, the present review documents and discusses the pharmacological effects of hypophyllanthin and its mechanisms of action that have been reported up-to-date. An insight into its pharmacological activities and mechanisms of action provides an understanding of its potential as a lead compound for the discovery of a drug candidate, especially for the development of therapies for inflammatory and immune-related diseases.

## 2 Methodology

The databases employed for data collection are mainly from Ovid-MEDLINE, Google Scholar, Scopus, Science Direct, and Elsevier databases from 1985 to 2022. Specific keywords used for the collection of data include “hypophyllanthin,” “*Phyllanthus*,” “immunosuppression,” “anti-inflammation,” “hepatoprotective,” “*in vitro* studies,” “*in vivo* studies.” The inclusion criteria for the papers are as follows: original research papers in English on pharmacological activities and mechanisms of action of hypophyllanthin; hypophyllanthin used can either be synthetic or an isolated natural compound; crude extracts of *Phyllanthus* species where hypophyllanthin contributes to bioactivities; *in vivo* and/or *in vitro* and/or *ex vivo* studies; toxicological information.

## 3 Isolation, qualitative and quantitative analyses of hypophyllanthin from *Phyllanthus* species

In efforts to investigate the biological and pharmacological activities of phyllanthin and hypophyllanthin, many workers reported the isolation of the lignans from various *Phyllanthus* species at > 95–98% purity by using column chromatography or preparative HPLC but the yields of the lignans obtained were mostly not recorded (Syamasundar et al., 1985; Joshi and Priya, 2007; Islam et al., 2008a; Islam et al., 2008b; Inchoo et al., 2011; Taesotikul et al., 2011; Kiran and Rao, 2013; Sukhaphirom et al., 2013; Parvathaneni et al., 2014a). A low yield of hypophyllanthin was obtained from *P. debilis* where 52.3 mg of the lignan was obtained from 40 g of ethanol extract (.13%) by using repeated silica gel column chromatography, preparative TLC and recrystallization technique (Chandrashekar et al., 2004). Hypophyllanthin was obtained at a higher yield at 4.16% w/w from *P. niruri* using column chromatography (Syamasundar et al., 1985). Yuandani et al. (2013) reported that fractionation of 10 g of the methanol extract of *P. amarus* collected from Malaysia and Indonesia by vacuum liquid chromatography on silica gel type H followed by repeated silica gel column chromatography led to isolation of 235.5–321.2 mg (2.35%–3.21%, respectively) of hypophyllanthin. The different amounts of hypophyllanthin isolated from the *P. amarus* were probably due to the origin of samples and the use of different separation techniques. Geographical location, soil condition and climate variability might also be factors contributing to the variable yields.

Qualitative and quantitative analyses of hypophyllanthin in the extracts of various *Phyllanthus* species obtained from different locations have been reported by many researchers. Tripathi et al. (2006) developed a rapid and precise high-performance thin-layer chromatography (HPTLC) for the quantification of phyllanthin and hypophyllanthin in the extracts of *P. amarus, P. urinaria, P. fraternus, P. maderaspatensis, P. virgatus*, and *P. debilis*. *P. amarus* was found to contain the highest concentrations of phyllanthin and hypophyllanthin. The other samples contained low concentrations of phyllanthin, and hypophyllanthin was not detected in any of them. Simultaneous detection of four lignans; phyllanthin, hypophyllanthin, phyltetralin, and niranthin; in various sections of *P. niruri* was established using a simple reverse phase HPLC approach with fluorescence detection (Murugaiyah and Chan, 2007a). The leaves of *P. niruri* were discovered to have a higher concentration of lignans than the other plant components, with phyllanthin being the most prevalent. The method developed was further used to conduct a pharmacokinetic study of hypophyllanthin in rats (Murugaiyah and Chan, 2007b). In this work, hypophyllanthin and the other lignans could be clearly recognised and detected in the rats’ plasma even after 10 h of oral lignan treatment. Another simultaneous quantitative measurement of four lignans (phyllanthin, hypophyllanthin, niranthin, and nirtetralin) from the leaves of four *Phyllanthus* species, namely *P. amarus*, *P. maderaspatensis*, *P. urinaria*, and *P. virgatus*, were developed using a standardised chiral TLC densitometric approach. The highest amount of hypophyllanthin was found in the extract of *P. amarus* (.383% w/w) while the compound was present at lower concentrations in *P. maderaspatensis* L (.013 w/w) and *P. virgatus* (.012 w/w) (Srivastava et al., 2008).

Hypophyllanthin together with phyllanthin and polyphenols, geraniin, gallic acid, corilagin ellagic acid, corilagin, and geraniin were qualitatively and quantitatively analyzed in the extracts of *P. amarus* and *P. urinaria* by using a validated reversed phase HPLC method (Jantan et al., 2014). *P. amarus* from Indonesia contained the highest amount of hypophyllantin (121.85 μg/mL) while plant from Malaysia contained the highest amounts of phyllanthin (103.5 μg/mL), gallic acid (163.3 μg/mL) and ellagic acid (601.29 μg/mL). A lower concentration (19.5 μg/mL) of hypophyllanthin was found in *P. urinaria* (Jantan et al., 2014). In another study, hypophyllanthin together with other main components (phyllanthin, ellagic acid, corilagin, geraniin, gallic acid) of 80% ethanol extract of *P. amarus* were identified in the extracts using validated reversed-phase HPLC methods. Quantitative determination of the major compounds by HPLC showed that hypophyllanthin was present at 108.11 μg/mL, which was higher than phyllanthin (99.49 μg/mL) (Ilongkovan et al., 2015).

A rapid qualitative and quantitative analyses of P*. amarus* extract using high-pressure liquid chromatography hyphenated with quadrupole time-of-flight mass spectrometer (HPLC/ESI-QTOF-MS/MS) led to the identification of 52 compounds out of which nine lignans were successfully characterized based on retention time, exact mass, molecular formula and their fragmentation patterns. Among the lignans, hypophyllanthin and phyllanthin were further quantified by UPLC/ESI-MS/MS method in multiple reaction monitoring (MRM) acquisition mode (Kumar et al., 2015). A validated chromatographic method was also used to quantitatively analyse the extracts of *P. niruri, P. emblica, P. amarus*, *P. fraternus*, and their herbal formulations. The ethyl acetate fraction of *P. amarus* was found to contain the highest concentrations of hypophyllanthin (29.40 mg/g) and phyllanthin (56.60 mg/g) (Kumar et al., 2017).

A rapid and sensitive HPLC–MS/MS method was developed and validated to simultaneously determine the concentrations of hypophyllanthin together with nirtetralin, phyllanthin and niranthin from *P. urinaria* in rat plasma in a pharmacokinetics study. The spectra showed the presence of sodium adduct [M + Na]^+^ ions as molecular ions for all lignans when utilising electrospray ionisation (ESI) positive ionisation mode. Hypophyllanthin did not produce protonated adduct [M + H]^+^ ions while the ions were produced at a low level for phyllanthin (Fan et al., 2015). In another study, metabolite changes of aqueous ethanol extracts of *P. niruri* were determined by using a proton nuclear magnetic resonance (^1^H-NMR)-based metabolomics approach. Freeze-dried sample of *P. niruri* extracted with 80% ethanol was found to contain the highest amounts of hypophyllanthin and phenolic compounds based on the loading plot of principal component analysis (PCA). The distinctive binned signals of hypophyllanthin were recorded at chemical shift 5.62 ppm based on ^1^H-NMR signals. The partial least-square (PLS) results showed that the phytochemicals, including hypophyllanthin were correlated with antioxidant and α-glucosidase inhibitory activities of the extracts, which were higher in the sample material extracted with 80% ethanol (Mediani et al., 2017). Table 1 shows the summary of qualitative or quantitative of hypophyllanthin in *Phyllanthus* species such as *P. amarus* (Somanabandhu et al., 1993; Tripathi et al., 2006; Srivastava et al., 2008; Inchoo et al., 2011; Ilangkovan et al., 2013; Kandhare et al., 2013; Sukhaphirom et al., 2013; Parvathaneni et al., 2014a; Kumar et al., 2015; Pradit et al., 2015; Kumar et al., 2017), *P. niruri* (Hussain et al., 1995; Murugaiyah and Chan 2006; Murugaiyah and Chan 2007a; Murugaiyah and Chan 2007b; Murugaiyah and Chan 2009; Jantan et al., 2014; Kumar et al., 2017; Mediani et al., 2017)*, P. urinaria* (Ilangkovan et al., 2013; Van Thanh et al., 2014; Fan et al., 2015)*, P. maderaspatensis* L. (Srivastava et al., 2008), and *P. virgatus* G. Forst. (Srivastava et al., 2008).

**TABLE 1:** Summary of qualitative and quantitative analysis of hypophyllanthin.

Species	Extract	Qualitative or quantitative analysis used to identify the presence of hypophyllanthin	References
*P. amarus* Schumm. and Thonn.	Hexane	• Melting point analysis	Somanabandhu et al. (1993)
		• Ultraviolet spectroscopy	
		• Infrared spectroscopy	
		• Mass spectrometry	
		• Proton nuclear magnetic resonance (^1^H-NMR)	
		• Carbon-13 nuclear magnetic resonance (^13^C-NMR)	
	Ethanol	• High-performance thin-layer chromatography (HPTLC) method with simple chiral densitometric	(Tripathi et al., 2006; Srivastava et al., 2008)
	Ethanol	• Infrared spectroscopy	Inchoo et al. (2011)
		• High-performance liquid chromatography (HPLC)	
	515Methanol	• Physicochemical properties analysis	Ilangkovan et al. (2013)
		• High-performance liquid chromatography (HPLC)	
		• Nuclear magnetic resonance (NMR)	
		• Mass spectrometry (ESI-MS)	
	Methanol	• High-Performance Liquid Chromatography (HPLC)	Kandhare et al. (2013)
	Ethanol	• High-pressure liquid chromatography hyphenated with quadrupole time-of-flight mass spectrometer (HPLC/ESI-QTOF-MS/MS)	Kumar et al. (2015)
		• Ultra-high performance liquid chromatography electrospray in tandem with mass spectrometry (UHPLC/ESI-MS/MS) in MRM mode	
	Ethanol	• Ultra-high performance liquid chromatography (UHPLC) in tandem with mass spectrometry (MS)	Kumar et al. (2017)
	Hexane	• Infrared spectroscopy	Sukhaphirom et al. (2013)
		• High-performance liquid chromatography (HPLC)	
	Hexane, 95% ethanol	• High-performance liquid chromatography (HPLC)	(Parvathaneni et al., 2014a; Pradit et al., 2015)
*P. niruri* L.	Methanol	• Melting point analysis	Hussain et al. (1995)
		• Ultraviolet spectroscopy	
		• Infrared spectroscopy	
		• Mass spectrometry	
		• Proton nuclear magnetic resonance (^1^H-NMR)	
		• Carbon-13 nuclear magnetic resonance (^13^C-NMR)	
	Methanol	• Ultraviolet spectroscopy	(Murugaiyah and Chan 2006; Murugaiyah and Chan 2009)
		• Infrared spectroscopy	
		• High-performance liquid chromatography (HPLC)	
		• Mass spectrometry	
		• Nuclear magnetic resonance (NMR)	
	Methanol	High-performance liquid chromatography (HPLC) with fluorescence detection	(Murugaiyah and Chan 2007a; Murugaiyah and Chan 2007b)
	80% Ethanol	• Ultraviolet spectroscopy	Jantan et al. (2014)
		• High-performance liquid chromatography (HPLC)	
	Ethanol	• Ultra-high performance liquid chromatography (UHPLC) in tandem with mass spectrometry (MS)	Kumar et al. (2017)
	Ethanol	• Proton nuclear magnetic resonance (^1^H-NMR)-based metabolomics	Mediani et al. (2017)
	95% Ethanol	• High-performance liquid chromatography (HPLC)	Cao et al. (2018)
		• Nuclear magnetic resonance (NMR)	
		• Mass spectrometry (ESI-MS)	
*P. urinaria* L.	Methanol	• Physicochemical properties analysis	Ilangkovan et al. (2013)
		• High-performance liquid chromatography (HPLC)	
		• Nuclear magnetic resonance (NMR)	
		• Mass spectrometry (ESI-MS)	
	Methanol	• Proton nuclear magnetic resonance (^1^H-NMR)	Van Thanh et al. (2014)
		• Carbon-13 nuclear magnetic resonance (^13^C-NMR)	
	80% Ethanol	• Liquid Chromatography with tandem mass spectrometry (HPLC-MS/MS)	Fan et al. (2015)
*P. maderaspatensis* L.	Ethanol	• High-performance thin-layer chromatography (HPTLC) with simple chiral densitometric	Srivastava et al. (2008)
*P. virgatus* G.Forst.	Ethanol	• High-performance thin-layer chromatography (HPTLC) with simple chiral densitometric	Srivastava et al. (2008)

## 4 Pharmacological activities and mechanisms of action

**FIGURE 3:**
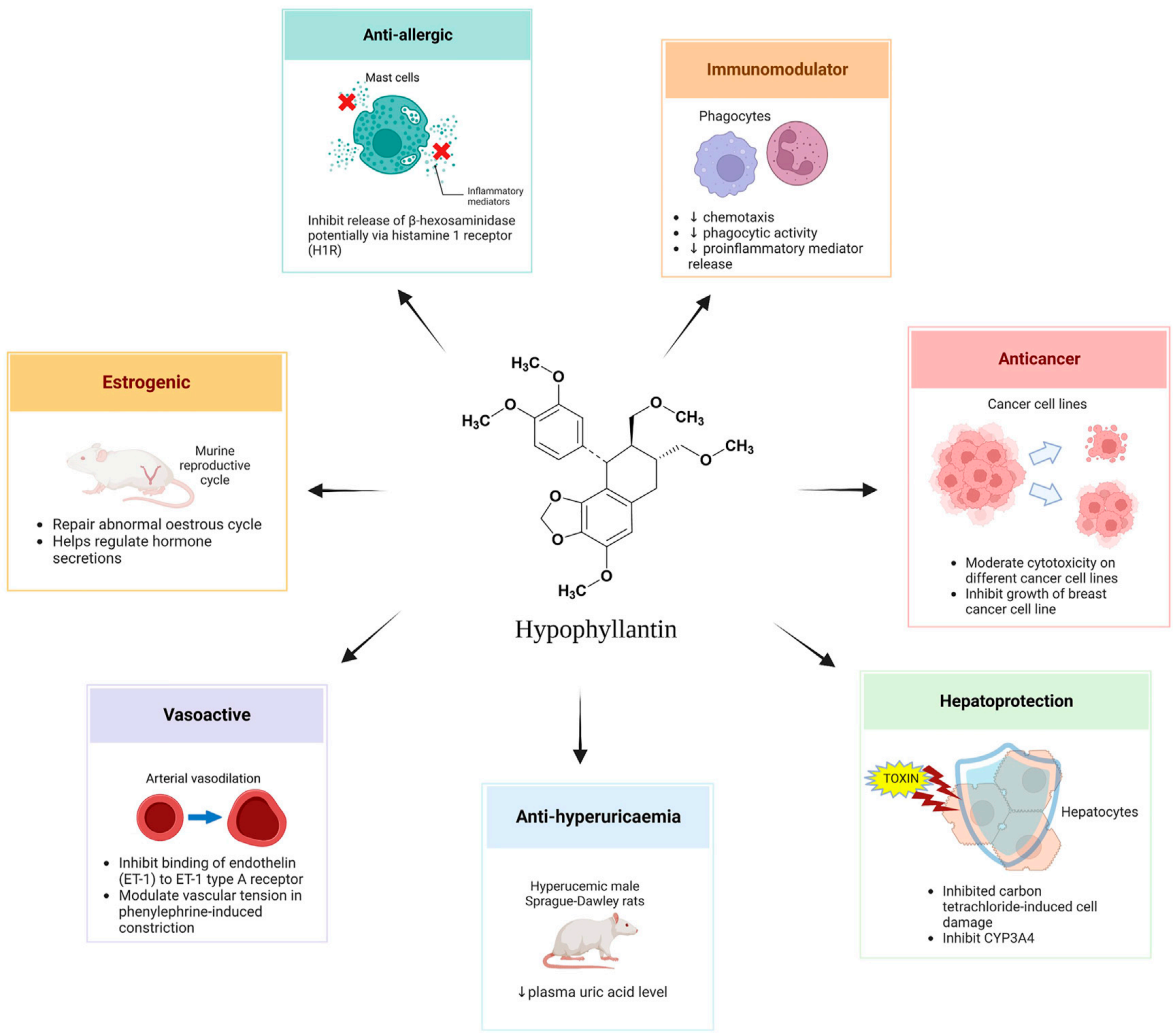
Pharmacological activities from hypophyllanthin from *in vitro* and *in vivo* studies.

In this review, up-to-date reports on the pharmacological activities and mechanisms of action of hypophyllanthin were gathered and critically analyzed. The bioactivities of extracts of *Phyllanthus* species where hypophyllanthin has partly been attributed to their activities have also been included in this discussion. *In vivo* and *in vitro* studies on the pharmacological activities of hypophyllanthin include immunomodulation, anti-inflammatory, anticancer, hepatoprotective, anti-hyperuricemic, vascular tension, and anti-allergic activities. Figure 3; Tables 2, 3 shows the various pharmacological activities of hypophyllanthin as a major component in the extracts of *Phyllanthus* species such as immunomodulation (Jantan et al., 2014; Harikrishnan et al., 2018a; Harikrishnan et al., 2018b; Harikrishnan et al., 2018c), anti-inflammatory (Rachmilewitz et al., 1989; Mali et al., 2011; Kandhare et al., 2013; Chopade and Sayyad, 2015; Ilangkovan et al., 2015; Pradit et al., 2015; Wu et al., 2019), anticancer (Somanabandhu et al., 1993; Islam et al., 2008b; Sukhaphirom et al., 2013; Parvathaneni et al., 2014b; Van Thanh et al., 2014), hepatoprotective (Syamasundar et al., 1985; Harish and Shivanandappa, 2006; Rajkapoor et al., 2008; Taesotikul et al., 2011; Srirama et al., 2012; Subramanian et al., 2019; Wahyuni et al., 2019), anti-hyperuricemic (Murugaiyah and Chan, 2006), vascular tension (Hussain et al., 1995; Inchoo et al., 2011), estrogenic (Islam et al., 2008a) and anti-allergic activities (Abd Rani et al., 2021).

**TABLE 2:** *In vitro* pharmacological activities of hypophyllanthin and extracts of *Phyllanthus* species containing hypophyllanthin.

*Phyllanthus* sp. *Name*	Country	Extract used/Synthetic source	Activity	Assay type/Type of model or cells	Mechanism of action (MOA)/Conclusion	References
*—*	United States	Synthetic: Chroma for hypophyllanthin	Immunomodulator (that leads to anti-inflammatory effect)	*In vitro* (Lipopolysaccharide (LPS)-induced U937 macrophages)	Inhibiting the activity of cyclooxygenase-2 (COX-2), interleukin-1 beta (IL-1β), tumour necrosis factor alpha (TNF-α) and prostaglandin E_2_ (PGE_*2*_) Hypophyllanthin also suppresses phosphorylation of c-Jun N-terminal kinase (JNK), extracellular signal-regulated kinase ERK and p38	Harikrishnan et al. (2018b)
*—*	United States	Synthetic: Sigma-Adrich for phyllanthin, hypophyllanthin, corilagin and niranthin	Anti-allergic	*In vitro* (Radioligand competition binding assay)	Hypophyllanthin showed stronger activity than chlorpheniramine as positive control and this indicates that hypophyllanthin might have potential as an antihistamine drug	Abd Rani et al. (2021)
*P. niruri* L.	Malaysia and Indonesia	80% ethanol	Immunomodulator	*In vitro* (Polymorphonuclear leukocytes (PMNs), and monocytes)	Inhibiting chemotaxis and phagocytosis as well as β2-integrin (CD18) expression on the PMNs and monocytes	Jantan et al. (2014)
				• Chemotaxis assay		
				• Phagocytic assay		
				• CD18 integrin expression assay		
				• Chemiluminescence assay		
*P. amarus* Schum. and Thonn and *P. urinaria* L*.*	Malaysia and Indonesia	Methanol	Anti-inflammation	*In vitro* (Polymorphonuclear leukocytes (PMNs) and monocytes)	Inhibiting phagocytosis process of the neutrophils and monocytes	Ilangkovan et al. (2015)
				• Chemotaxis assay		
				• Phagocytic assay		
				• Chemiluminescence assay		
*P. amarus* Schum. and Thonn.	Thailand	90% ethanol	Chondroprotective	*In vitro* (Porcine articular cartilage explant cultures)	Hypophyllanthin contributed in preventing the degradation of the cartilage that usually occur in osteoarthritis	Pradit et al. (2015)
*P. amarus* Schum. and Thonn.	Thailand	Hexane	Anti-tumour/Cytotoxic in Multidrug-Resistant Cells	*In vitro* (human epithelial cancer cells: KB-V1 and KB-3 cell line)	Hypophyllanthin demonstrated cytotoxic action in KB-V1 cell only	Somanabandhu et al. (1993)
*P. urinaria* L.	Vietnam	Methanol	Cytotoxic	*In vitro* (Murine macrophage (J774) and Chinese hamster vary (CHO) cell lines)	Isolated hypophyllanthin exhibited moderate cytotoxic activity on both J774 and CHO cell lines	Van Thanh et al. (2014)
*P. amarus* Schum. and Thonn.	India	Hexane	Anti-tumour (Breast *Cancer*)	*In vitro* (breast cancer cell lines: MCF-7 and MDA-MB-231 cell lines using 3-(4,5-dimethylthiazol-2-yl)-2,5-diphenyl-2H-tetrazolium bromide (MTT) assay)	Hypophyllanthin showed inhibitory action on breast cancer cell lines	Parvathaneni et al. (2014a)
*P. amarus* Schum. and Thonn.	N/A	Hexane	P-gp transporter inhibitor	*In-vitro* (Human colon cancer: Caco-2 cells)	Hypophyllanthin could bound to P-glycoprotein (P-gp) binding site and reversibly inhibited its fuction as drug efflux pump. However, this action might not be effective with long-term treatment. Besides, it also did not affect the multidrug resistance protein 2 (MRP-2) activity significantly	Sukhaphirom et al. (2013)
*P. niruri* L.	N/A	Hexane	Hepatoprotective	*In vitro* (Primary cultured rat hepatocytes)	The extract containing hypophyllanthin showed inhibited hepatotoxicity in the rat hepatocytes that were treated with toxic substances	Syamasundar et al. (1985)
*P. niruri* L.	India	Methanol and aqueous	Hepatoprotective and Antioxidant	*In vitro* (2,2-diphenyl-1-picryl-hydrazyl-hydrate (DPPH) assay)	The extract containing hypophyllanthin exhibited liver protective action by its antioxidant properties	(Harish and Shivanandappa, 2006)
*P. niruri* L.	China	95% Ethanol	Anticancer	*In vitro* (human lung cancer cell line A549, hepatic cancer cell line SMMC-7721, gastric cancer cell line MGC-803)	Hypophyllanthin exhibited potent anti-cancer against A549, SMMC-7721 and MGC-803, respectively	Cao et al. (2018)
*P. niruri* L.	Indonesia	Ethanol	Hepatoprotective and antiviral	*In vitro* (immortal cell lines (Huh 7it)	The extract containing hypophyllanthin exhibited antiviral action against hepatitis C virus (HCV) which can infect hepatocyte cells of an organism	Wahyuni et al. (2019)
*P. niruri* L.	Malaysia	Methanol Hexane	Antihyperuricemic	*In vitro* (Xanthine oxidase assay)	The extract containing hypophyllanthin and other lignans had reduced the uric acid level in the plasma of experimental subjects. The extract also inhibited xanthine oxidase enzyme	Murugaiyah and Chan, (2009)
*P. niruri* L.	India	Methanol	Vasodilation	*In vitro* (Rat thoracic aortic smooth muscle cells culture)	Hypophyllanthin showed vasodilation activity by inhibiting the activity of endothelin (vasoconstrictor) specifically on endothelin A-type receptor	Hussain et al. (1995)
*P. amarus* Schum. and Thonn.	N/A	Hexane	Inhibiting vasoconstriction	*In vitro* (Aortic tissues of male Wistar rats)	Hypophyllanthin functioned as weak inhibitor on vasoconstriction in the muscle	Inchoo et al. (2011)
*P. amarus Schum. and Thonn.*	Malaysia	80% ethanol	Anti-allergic	*In vitro* (Radioligand competition binding assay)	Hypophyllanthin showed stronger activity than chlorpheniramine as positive control and this indicates that hypophyllanthin might have potential as an antihistamine drug	Abd Rani et al. (2021)

*N/A: no available information.

**TABLE 3:** *In vivo* pharmacological activities of hypophyllanthin and extracts of *Phyllanthus* species containing hypophyllanthin.

*Phyllanthus* sp. *Name*	Country	Extract used/Synthetic source	Activity	Assay type/Type of model or cells	Mechanism of action (MOA)/Conclusion	References
*P. amarus* Schum. and Thonn.	India	Standardized methanolic extract of 2.5% phyllanthin and hypophyllanthin	Anti-inflammation	*In vivo* (Male Wistar rats)	Reducing musle pain by inhibiting prostaglandin E_2_ (PGE_2_)	Chopade and Sayyad, (2015)
*P. amarus* Schum. and Thonn.	India	Methanol standardized extract 2.5% phyllanthin and hypophyllanthin	Anti-inflammation (ulcerative colitis)	*In vivo* (Male Swiss albino mice and male Wistar rats)	Hypophyllanthin contributed to anti-inflammatory action against ulcerative colitis that is manifested by *P. amarus*. The presence of hypophyllanthin aids in reduction of colon weight to length ratio as well as colon wet weight of experimental subjects which were ulcerative colitis-induced	Kandhare et al. (2013)
*P. amarus* Schum. and Thonn.	India	Aqueous standardized extract 2.5% phyllanthin and hypophyllanthin	Anti-inflammation/Anti-arthritic	*In vivo* (Female Wistar rats)	Hypophyllanthin in the extract contributed to reduction in inflammation that can be observed on tibiotarsal joint in histopathological study	Mali et al. (2011)
*P. amarus* Schum. and Thonn.	N/A	Methanol	Anti-inflammation/Anti-asthmatic	*In vivo* (Sprague-Dawley rats)	Inhibiting inflammatory mediators infiltration and alleviate T helper type-2 (Th2) actions through amelioration of oxidonitorsative stress	Wu et al. (2019)
*P. amarus* Schum. and Thonn.	India	Methanol	Anti-tumour (Ehrlich Ascites Carcinoma)	*In vivo* (Male Swiss albino mice)	Hypophyllanthin suppressed tumour volume, viable cell count and increased survival time in cancer-bearing experimental subjects	Islam, Selvan, et al. (2008b)
*P. amarus* Schum. and Thonn.	India	Hexane	Anti-tumour (Breast *Cancer*)	*In-vivo* (Sprague-Dawley rats)	Hypophyllanthin showed inhibitory action on breast cancer cell lines	Parvathaneni et al. (2014b)
*P. amarus* Schum. and Thonn.	T’hailand	Ethanol and aqueous	CYP3A4 inhibitor	*In vivo* (Human microsomal liver)	Hypophyllanthin inhibited human liver microsome, cytochrome P450 3A4 (CYP3A4) that is classified under CYP450, in which most of the drugs are also metabolised here	Taesotikul et al. (2011)
*P. amarus* Schum. and Thonn.	N/A	N/A	Estrogenic	*In vivo* (Female Wistar rats)	Hypophyllanthin regulated the estrous cycles that were disrupted due to exposure to toxic substance	Islam, Naskar, et al. (2008a)
*P. niruri* L.	Malaysia	Methanol	Antihyperuricemic	*In vivo* (Male Sprague-Dawley rats)	Hypophyllanthin contributed to the plant’s action as antihyperuricemic agent by mainly decreasing the level of uric acid in the plasma	Murugaiyah and Chan, (2006)
*P. niruri* L.	Malaysia	Methanol Hexane	Antihyperuricemic	*In vivo* (Male Sprague-Dawley rats)	The extract containing hypophyllanthin and other lignans had reduced the uric acid level in the plasma of experimental subjects. The extract also inhibited xanthine oxidase enzyme	Murugaiyah and Chan, (2009)

*N/A: no available information.

### 4.1 Immunomodulating activity

Innate immune responses involve phagocytes such as polymorphonuclear neutrophils (PMNs), monocytes, macrophages, and dendritic cells. Upon exposure to pathogens, these immune cells are activated and are able to transmigrate from the peripheral blood to the infected area involving endothelial adhesion, transmigration, recognition of pathogen *via* various pathogen recognition receptors (PRRs), and ultimately engulfing the pathogen for intracellular destruction. Hypophyllanthin has been demonstrated in many *in vitro* and *in vivo* experiments to possess immunomodulating activity on phagocytes. The methanol extracts of *P. amarus* and *P. urinaria* and their bioactive isolates, phyllanthin and hypophyllanthin, have showed inhibitory effects on the chemotaxis, phagocytosis and reactive oxygen species (ROS) production of human phagocytes in a dose-dependent manner. Hypophyllanthin and phyllanthin isolated from the methanol extracts of *P. amarus* and *P. urinaria* exhibited relatively strong and dose-dependent effect on polymorphonuclear leukocyte (PMNs) chemotaxis, with IC_50_ values of 7.6 and 10.2 μM, respectively, which were slightly higher than the value for the positive control, ibuprofen (6.8 μM). Phyllanthin and hypophyllanthin also demonstrated marked suppressive activity on the oxidative burst in whole blood with IC_50_ values of 8.8 and 8.4 μM, respectively, which were lower than that of aspirin (12.2 μM) (Yuandani et al., 2013). In another study, the 80% ethanol extracts of *P. amarus and P. urinaria* and their phenolic compounds and lignans including hypophyllanthin and phyllanthin were evaluated for their effects on chemotaxis, β2-integrin (CD18) expression, phagocytosis and chemiluminescence of human phagocytes. The plant extracts regulated the immune regulatory action through various pathways as they exhibited strong inhibitory activity on ROS generation and chemotactic activity of phagocytes stimulated by different stimuli. Hypohyllanthin and phyllanthin were found to exhibit potent inhibitory action on both phagocytic and CD18 expression of phagocytes. At 50 μg/mL, hypophyllanthin was found to exhibit a high engulfment inhibitory activity with a percentage of phagocytizing cells of 49.11% and 64.6% for PMNs and monocytes, respectively. At similar concentrations, hypophyllanthin reduced the CD18 expression in PMNs and monocytes to 74.7% and 74.4%, respectively. Hypophyllanthin together with other major components of the extracts especially geraniin, corilagin, phyllanthin could be the major contributors to the strong immunomodulatory effect of the plant extracts as they were able to modulate the innate response of phagocytes at different steps (Jantan et al., 2014).

**FIGURE 4:**
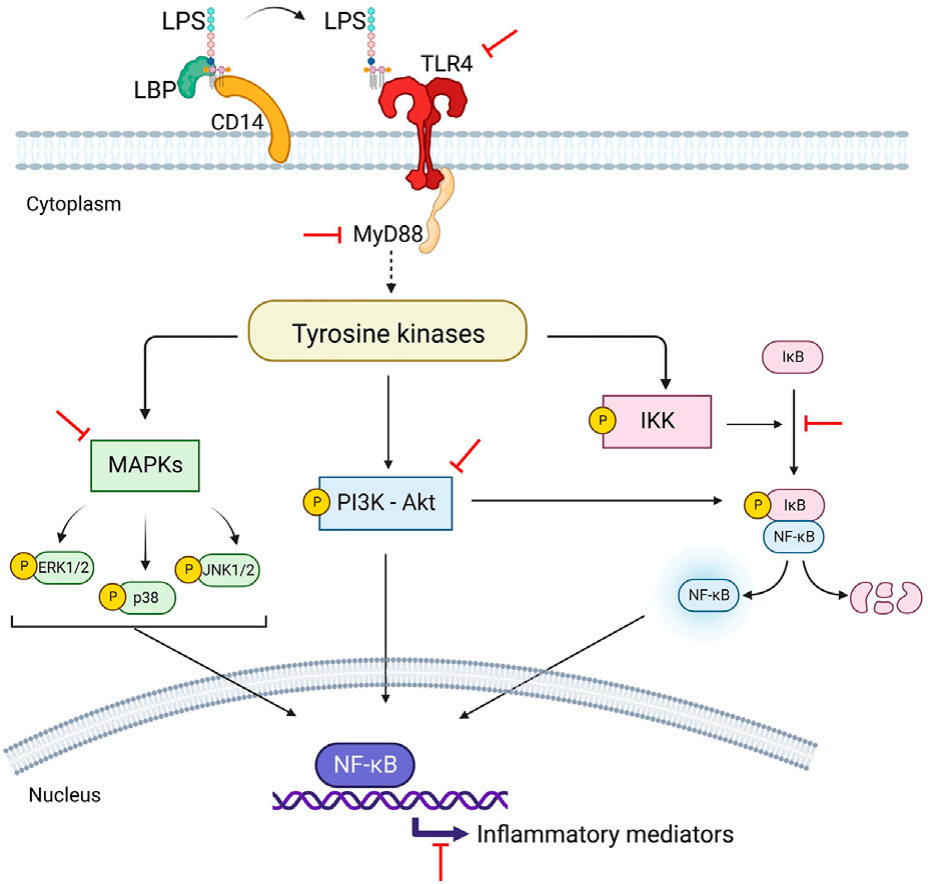
Hypophyllanthin-mediated inhibition of lipopolysaccharide (LPS)-dependant NF-κΒ, MAPK and P13K-Akt signaling pathways in human macrophages.

The immunosuppressive effects of 80% ethanol extract of *P. amarus* and its bioactive lignans including hypophyllanthin, phyllanthin and niranthin on pro-inflammatory mediators release *via* in nuclear factor-kappa B (NF-кB), mitogen activated protein kinase (MAPK) and phosphatidylinositol 3-kinase/Akt (PI3K-Akt) signaling activation in lipopolysaccharide (LPS)-induced U937 human macrophages have been documented (Harikrishnan et al., 2018a; Harikrishnan et al., 2018b; Harikrishnan et al., 2018c). Hypophyllanthin significantly downregulated the gene expression and protein levels of cyclooxygenase-2 (COX-2) as well as the downstream signaling products of prostaglandin E_2_ (PGE_2_), tumor necrosis factor-alpha (TNF-α), and interleukin 1 beta (IL-1β) by suppressing the initiation of MAPKs, NF-κB, and protein kinase B (Akt) in a dose-dependent manner. Hypophyllanthin demonstrated the ability to suppress IκB degradation and the inhibitors of IkB kinases (Ikkα/β), kappa B (IκB) and NF-κB phosphorylation. Hypophyllanthin also inhibited the phosphorylation of extracellular signal-regulated kinase (ERK), c-Jun N-terminal kinase (JNK) and p38. The results indicated that hypophyllanthin has the potential to be developed as an anti-inflammatory agent targeting the NF-κB, MAPK, and PI3K-Akt pathways (Harikrishnan et al., 2018c). Figure 4 depicts the immunosuppressive effects of hypophyllanthin on the immune responses by targeting NF-κB, MAPK, and protein kinase B (Akt) pathways.

There are many studies on the *in vivo* immunomodulatory effects of *Phyllanthus* species where hypophyllanthin was identified as one of the bioactive compounds contributing to the immunomodulating effects on the immune responses (Ilangkovan et al., 2015; Ilangkovan et al., 2016; Wu et al., 2019) For example, the suppressive effect of *P. amarus* extract (50, 100, and 200 mg/kg, p. o.) treatment for 28 days against ovalbumin (OVA)-induced experimental airway hyperresponsiveness (AHR) in Sprague-Dawley rats has been documented. The results showed that the extract significantly (*p* < .05) ameliorated OVA-induced increase in oxido-nitrosative stress (superoxide dismutase (SOD), glutathione (GSH), malondialdehyde (MDA), and nitric oxide (NO) levels and immunoglobulin E (IgE) (total and OVA-specific). *P. amarus* extract also significantly (*p* < .05) attenuated the elevated immune-inflammatory makers (heme oxygenase-1 (HO-1), TNF-α, IL-1β, and transforming growth factor beta-1 (TGF-β1), oxido-nitrosative stress (nuclear factor erythroid 2–related factor 2 (Nrf2) and inducible nitric oxide synthase (iNOs)) and T helper type 2 (Th2) cytokines (IL-4 and IL-6) levels, as determined by reverse transcription polymerase chain reaction (RT-PCR) analysis (Wu et al., 2019). The authors concluded that the presence of phyllanthin, and hypophyllanthin in the extract played an important role in ameliorating AHR conditions in OVA-induced experimental rats by reducing pro-inflammatory molecules and preventing airway remodeling.

Ilangkovan et al. (2015) investigated the modulating effect of the standardized 80% ethanol extract of *P. amarus* (100–400 mg/kg) on various cellular immune parameters in Wistar-Kyoto rats. The extract was administered to the rats for 14 days and was found to attenuate *E. coli* engulfment, Mac-1 expression, T- and B cell proliferation, CD4^+^ and CD8^+^ T cell subsets, and serum cytokine production in leukocytes isolated from treated/non-treated rats. At a dose of 400 mg/kg, there was a significant decrease (*p* < .01%) in the percentage expression of CD4^+^ and CD8^+^ in splenocytes and in serum cytokines of T helper (Th1) (interleukin-2 (IL-2) and interferon gamma (IFN-γ)) and Th2 (IL-4). In another study, Ilangkovan et al. (2016) determined the suppressive effects of daily treatment of a standardized extract of *P. amarus* (50, 100 and 200 mg/kg) for 14 days in Balb/C mice on the myeloperoxidase activity (MPO), NO release, macrophage phagocytosis, swelling of a footpad in delayed-type hypersensitivity (DTH), and serum immunoglobulins, ceruloplasmin and lysozyme levels. Hypophyllanthin (108.11 μg/mL) present as one of the major compounds in the extract contributed partly to the immunosuppressive effect on the cellular and humoral immune responses in the mice. The standardized 80% ethanol extract of *P. amarus* was also evaluated for its protective effects on LPS-stimulated neuroinflammation and non-spatial memory impairment in rats (Alagan et al., 2019). The rats were effectively protected from LPS-induced memory impairment after daily administration of the extract at 200 and 400 mg/kg for 14 and 28 days. The extract also significantly (*p* < .05) decreased the release of IL-1β, TNF-α, iNOS, NO levels, CD11b/c integrin expression, and synaptophysin immunoreactivity in the brain tissue as compared with those in the LPS-challenged group. The protective effects of the extract on LPS-induced non-spatial memory impairment and neuroinflammation might partly been attributed to hypophyllanthin, which was present at 95.37 μg/mL in the extract.

Based on the *in vitro* studies, hypophyllantin has demonstrated substantial immunomodulatory activity and was comparable to the controls used. However, the observed effects from the *in vivo* models were associated with the presence of hypophyllantin in the plant extracts used. Therefore, in addition to the reported immunomodulatory effects from *in vitro* assays, we can further explore the ability of hypophyllantin in modulating immune response by using animal models treated with the bioactive compound itself to help further analyze its systemic role.

### 4.2 Anti-inflammatory activity

The presence of chronic inflammation during abnormal immune responses would lead to a variety of negative consequences. Even if the immune response is an essential and vital method of protecting a host from harmful stimuli, such as the invasion of pathogens, it would lead to the release of a number of pro-inflammatory mediators such as interferon (IFN), interleukin 1 beta (IL-1β), tumour necrosis factor alpha (TNF-α), prostaglandin E_2_ (PGE_2)_, cyclooxygenase-2 (COX-2), nitric oxide (NO), and inducible nitric oxide synthase (iNOS) that are predisposed to cause inflammation. It was observed that the excessive release of these pro-inflammatory mediators may contribute to the development of chronic inflammation-related illnesses (Harikrishnan et al., 2018a).

The *P. amarus* extract also has the potential to act as a chondroprotective agent that prevents cartilage degeneration. This activity may be associated with the anti-inflammatory and anti-arthritic properties of hypophyllanthin. Hence, utilising the extract containing hypophyllanthin might provide an alternative treatment for osteoarthritis since cartilage degradation is the key event in the disease process. The protective activity on the cartilage tissue exhibited by hypophyllanthin was investigated in an *in vitro* experiment (Pradit et al., 2015). Hypophyllanthin inhibited the effect of IL-1 on the release of sulphate glycosaminoglycans (s-GAGs) in a dose-dependent manner. As a result, the matrix content of uronic acid and proteoglycans could be maintained given that hypophyllanthin had restored s-GAGs release to its normal level—a necessary process for maintaining the flexibility and viscoelasticity of tissues. The results revealed that hypophyllanthin treatment at a dose of 100 µM resulted in 100% s-GAGs release. A similar result was observed in the normal group. This outcome reflected that 100 µM hypophyllanthin treatment could normalise and counteract the action of 25 ng/mL IL-1β on the s-GAGs release. In comparison to the positive controls, 100 µM hypophyllanthin treatment recorded a stronger activity than 50 ug/mL of diacerein (125% of s-GAGs release), but slightly weaker relative to 10 µM of sesamin (75% of s-GAGs release). Moreover, 100 µM of hypophyllanthin treatment prevented the reduction of uronic acid contents in the cartilage, demonstrating similar results as in the normal group. Hence, 100 µM of hypophyllanthin treatment exhibited the strongest activity compared to both positive controls (10 µM of sesamin (80% of control) and 50 ug/mL of diacerein (90% of control)). For the proteoglycan content assay, the activity of hypophyllanthin treatment (100 µM) was similar to that of diacerein (50 ug/mL) but slightly lower than that of 10 µM of sesamin. Hypophyllanthin at a concentration of 100 μM significantly reduced the activity of matrix metalloproteinase-2 (MMP-2), which was able to degrade the matrix of the cartilage.

Many *in vivo* studies on the anti-inflammatory effects of *Phyllanthus* species where hypophyllanthin was identified as one of the bioactive compounds contributing to the effects in various animal models have been reported. Chopade and Sayyad, (2015) reported that the administration of lignans (phyllanthin and hypophyllanthin) and tannin (corilagin) rich extract of *P. amarus* (100, 200, and 400 mg/kg) significantly (*p* < .01) decreased the severity of carrageenan-induced thermal hyperalgesia in a model of chronic musculoskeletal inflammatory pain. The standardised methanol extract, comprised of phyllanthin and hypophyllanthin significantly (*p* < .05) reduced mechanical hyperalgesia and at doses of 200 and 400 mg/kg, the extract was as effective as the standard compound, aclofenac. The antihyperalgesic and anti-inflammatory effects of the extract elevated thermal and mechanical threshold, which was supported by histopathological observations along with lowered concentration of PGE_2_ in muscle exudates to a level comparable to the standard (*p* < .01). It was suggested that inhibition of chronic muscular inflammation by the extract might be due to the presence of bioactive constituents like hypophyllanthin, phyllanthin, and corilagin,

The protective effect of an aqueous extract of *Phyllanthus amarus* standardised to hypophyllanthin and phyllanthin in acetic acid-induced intracolonic ulcerative colitis in male Wistar rats was reported by Kandhare et al. (2013). The extract at 100 and 200 mg/kg orally administered to the rats provided protective effects against inflammation, oxidation, and apoptotic effect in a model of inflammatory bowel disease. There was a significant increase in the contents of glutathione (GSH) and superoxide dismutase (SOD), inhibition of myeloperoxidase (MPO) activity, a decrease of malondialdehyde (MDA) and a reduction in NO as well as TNF-α production. The extent of deoxyribonucleic acid (DNA) fragmentation was also found to be significantly reduced. The reduction in the occurrence of these essential mechanisms, i.e., inhibiting the infiltration of phagocytes, the generation of pro-inflammatory chemicals, and DNA damage may address ulcerative colitis. The effect of *P. amarus* extract treatment on the rat colon wet weight which is a well-established indicator of inflammation, and colon weight-to-length ratio was also evaluated. Pretreatment with the extract at 100 and 200 mg/kg gave a positive impact by significantly reducing the rat’s colon wet weight to 1.99 ± .07 g (*p* < .01) and 1.55 ± .09 g (*p* < .001), respectively, compared to untreated subjects with higher colon weight (2.69 ± .12 g). The reduction in colon weight was comparable to that of prednisolone, the positive control, which reduced the colon weight of the subjects to 1.39 ± .11 g (*p* < .001) at a dose of 2 mg/kg. The improvement in ulcerative colitis was also reflected by the low value of colonic weight:length ratio, which was achievable with the treatment of *P. amarus* extract. The ratio value for the untreated ulcerative colitis-induced rats was significantly higher (.21 ± .011 g/cm) compared to that of the normal rats (.08 ± .008 g/cm) and those treated with 100 mg/kg (.15 ± .008 g/cm) and 200 mg/kg (.12 ± .011 g/cm) of *P. amarus* extract. The colon weight to length ratio of the group treated prednisolone was .10 ± .009 g/cm. The results suggested that hypophyllanthin present in the extract might contribute significantly to the potential curative effect of the extract for ulcerative colitis.

Inflammation plays an important role in the development of rheumatoid arthritis, which is also a chronic condition. *P. amarus* standardised aqueous extract (phyllanthin and hypophyllanthin as chemical markers) demonstrated anti-inflammatory activities by considerably reducing arthritis symptoms in Wistar rats treated with Freund’s complete adjuvant (FCA) (Mali et al., 2011). The anti-inflammatory action of the extract was reflected by a significant and dose-dependent decrease in paw volume, arthritic index values, and joint diameter, as well as an increase in the threshold for mechanical hyperalgesia and nociceptive pain. The rats’ paw volume decreased consistently following the treatment with 200 and 400 mg/kg of aqueous standardised extract for 28 days. The groups administered with 200 and 400 mg/kg of the extract had paw volumes of 1.8 and .9 mL, respectively, compared to the positive control, indomethacin, which recorded a paw volume of less than .5 mL at 10 mg/kg. In addition, the treated groups and indomethacin demonstrated improved arthritis scores. The group receiving indomethacin exhibited the lowest arthritis score (less than .5), followed by those administered with 400 mg/kg (approximately 1) and 200 mg/kg (approximately 1.8), respectively. Histopathological analysis revealed that the groups administered with 400 mg/kg extract and 10 mg/kg of indomethacin exhibited normal connective tissues on the tibiotarsal joint and a lack of necrosis and lymphocytic infiltration of the tissues. The authors suggested that hypophyllanthin and phyllanthin were predominantly responsible for the anti-arthritic activity of the standardised extract. The extract was shown to be safe in the acute toxicity investigation as the standardized extract posed no toxicity to the subjects, despite using an extremely high dose of 2000 mg/kg. The serum levels of alanine aminotransferase (ALT) and aspartate aminotransferase (AST) decreased significantly in rats’ hepatocytes after treatment with the standardized extract. The results suggested that the extract with hypophyllanthin and phyllantin as the major compounds has potential to be developed into agent to treat arthritic condition.

The findings of the abovementioned studies suggest that the anti-inflammatory activities of hypophyllanthin, phyllantin and corilagin were attributable to their capacities to prevent cartilage degeneration, reduce excessive hyperalgesia, oxidative stress and inflammation responses. These beneficial effects of the compounds were comparable to those of clinically used positive control drugs. They also have great potential in disease management for ulcerative colitis and rheumatoid arthritis as evidenced in rat animal models. However, detailed mechanistic insights focusing on its molecular pathways should be further studied to better understand its mechanism of action.

### 4.3 Anticancer activity

Somanabandhu et al. (1993) conducted one of the early investigations on the cytotoxic potential of hypophyllanthin. This study used both cultured leukemia P-388 cells and a variety of human tumour cell lines. The researchers found that hypophyllanthin did not demonstrate any cytotoxic activities without the presence of vinblastine, a vinca alkaloid that has already been established and used extensively as a pharmacotherapy agent in cancer. Nevertheless, the combination of hypophyllanthin with vinblastine exhibited reasonable cytotoxic action on the human epithelial (KB-V1) cancer cell line with an ED_50_ value of 3.8 μg/mL. Additionally, the cytotoxic effect of hypophyllanthin was restricted to specific types of tumour cell lines while no effect was observed in the drug-sensitive KB-3 cell line. A study conducted by Van Thanh et al. (2014) reported that hypophyllanthin was one of the lignans with remarkable cytotoxic activity. Moderate cytotoxic activity of hypophyllanthin could be observed in Chinese hamster ovary (CHO) and murine macrophage (J774) cell lines. The IC_50_ values of hypophyllanthin was 16.88 and 21.25 µM for CHO and J774 cell lines, respectively. These IC_50_ values indicated that hypophylanthin was more potent as compared to other lignans with IC_50_ values greater than 50 μM. Non-etheless, hypophyllanthin was considered to possess moderate cytotoxic activity since the positive control, cycloheximide, recorded much lower IC_50_ values in CHO and J774 cell lines corresponding to 8.07 and 6.00 µM, respectively.

Sukhaphirom et al. (2013) investigated the inhibitory effect of hypophyllanthin and phyllanthin on the inhibition of P-glycoprotein (P-gp) and multidrug resistance protein 2 (MRP 2) in caco-2 cells. P-gp is one of the vital membrane transporters that are present in most cells and plays a key role in the occurrence of MDR. It participates in the transportation of drugs used in chemotherapy and other substances across the plasma membranes of a specific cell. The researchers found that hypophyllanthin exhibited a moderately potent inhibitory effect on P-gp function. Furthermore, the fluorescence of calcein revealed that 100 M of hypophyllanthin inhibited P-gp function with the same activity as 100 M of verapamil. In contrast, hypophyllanthin recorded no discernible effect on the inhibition of MRP2 activity when compared to the positive control, indomethacin (500 μM). Additionally, the function of P-gp was not inhibited by prolonged exposure of the subject cells to hypophyllanthin. The experimental data also disclosed the same calcein-AM readings (approximately 3-fold) on days 2 and 7 of the experiment. Therefore, hypophyllanthin likely inhibited P-gp activity reversibly and directly but did not affect MRP2 activity.

Parvathaneni et al. (2014a) also investigated the potential anti-tumour effect of hypophyllanthin isolated from the aerial parts of *P. amarus.* The researchers assessed the effect of hypophyllanthin on the proliferation of two separate breast cancer cell lines, MCF-7 and MDA-MB-231 by using the 3-(4,5-dimethylthiazol-2-yl)-2,5-diphenyl-2H-tetrazolium bromide (MTT) assay and the inhibitory effect of hypophyllanthin on the growth of mammary tumours in Sprague-Dawley rats induced with N-methyl-N-nitrosourea (MNU). The *in vitro* cytotoxicity study revealed that hypophyllanthin inhibited the development of the breast cancer cells in a dose-dependent manner with IC_50_ of 35.18 ± 1.48 μg/mL, but at a lower potency than phyllanthin. The oral administration of hypophyllanthin at doses of 5 and 10 mg/kg reduced the incidence of mammary cancer among the subjects. The incidence rate of cancer in the untreated MNU-induced group was 85.7%, which was much higher than that of the group treated with 5 and 10 mg/kg hypophyllanthin at an incidence rate of 62.5% and 50.0%, respectively. Moreover, the effect of hypophyllanthin was also evaluated based on the total tumour mass. The group treated with 5 and 10 mg/kg of hypophyllanthin recorded a total tumour mass of 12.82 and 12.06 g, respectively, a significant (*p* < .01) decrease compared to the untreated group. Despite such a remarkable change lacking upon increasing the dose of hypophyllanthin, the total tumour masses were still much lower than the MNU-induced group (35.85 g). In contrast, the tumour mass in the group treated with 2 mg/kg of tamoxifen was only 4.67 g (*p* < .01), thereby indicating that hypophyllanthin may have a moderate level of activity.

Cao et al. (2018) also reported the anticancer effect of hypophyllanthin and phyllanthin isolated from 95% ethanol *P. niruri* against three human cancer cell line, lung cancer A549 cells, hepatic cancer SMMC-7721 cells and gastric cancer MGC-803 cells, using the MTT assay with cis-platinum as positive control. The results demonstrated that hypophyllanthin exhibited potent anti-cancer effect, with IC_50_ values of .228, .181, .184 mM against A549 cells, SMMC-7721 cells, and MGC-803 cells, respectively, whereas phyllanthin exhibited significant anti-cancer activity only against SMMC-7721 cells, with an IC_50_ value of .276 mM. In addition, a Molecular Operating Environment (MOE)-docking analysis revealed that hypophyllanthin was completely bound to the active pockets of 4ZSE (lung cancer-related protein), 3QBY (hepatic cancer-related protein) and 4OUM (gastric cancer-related protein) with the best docking score of −14.0546, −12.8176 and −10.841 kcal/mol, respectively. Therefore, the researchers concluded that the protein 4ZSE, 3QBY, and 4OUM may be protein targets of hypophyllanthin for anticancer activities and might be potential active site protein (Cao et al., 2018).

Islam et al. (2008b) performed an *in vivo* study in which hypophyllanthin and phyllanthin isolated from the *P. amarus* exhibited anti-tumour activities against Ehrlich ascites carcinoma (EAC), which were transplanted intraperitoneally in the male Swiss albino mice. A mixture of hypophyllanthin and phyllanthin (1:1) was administered to the mice *via* oral route at increasing doses of 25, 50 and 100 mg/kg body weight. The beneficial effect of the lignan mixture was reflected in the reduced tumour volume and improved haematological parameters, such as haemoglobin and haematocrit levels. After 2 weeks of EAC induction, the experimental subjects recorded an increased white blood cell count but the red blood cell count was significantly reduced, resulting in anaemia. On the other hand, the lignan mixture demonstrated effective protective actions in preserving normal haematological parameters. The cancer-bearing mice treated with the lignan sample at doses of 25, 50, and 100 mg/kg restored the haematological parameters to or approaching normal values compared to the anaemic condition induced by the malignant condition. The anti-tumour activities of the lignans were also determined by observing the mean survival time and the percentage of increased life span (% ILS). The findings revealed that hypophyllanthin increased the mean survival time to 32.52 days (% ILS = 69.02), which was lower compared to that of phyllanthin at 45.42 days (% ILS = 136.07) and 5-fluorouracil, the standard drug in chemotherapy treatment (mean survival time = 51.38; % ILS = 167.05). Despite both lignans demonstrating beneficial biological actions at doses of 25 and 50 mg/kg, a short-term toxicity test revealed that at a dose of 100 mg/kg, mice displayed symptoms of toxicity such as inactivity, loss of appetite, hypothermia, and erected hairs. This result indicates that the lignans may be toxic to an organism if administered in excessive doses.

The haematological parameters were also addressed in the *in vivo* study conducted by Parvathaneni et al. (2014a), similar to the prior work by Islam et al. (2008b). The number of red blood cells (RBCs), platelets (PLTs), and haemoglobin (Hb) decreased significantly (*p* < .05) in untreated cancer subjects, which might have contributed to the onset of anaemia. On the other hand, the number of white blood cells (WBCs) increased in the untreated subjects, which suggests that the group was indeed affected by the condition. All haematological parameters affected by breast cancer induction were significantly restored after initiating treatment with hypophyllanthin. This result was consistent with the expected impact of the standard drug. All haematological indicators were significantly (*p* < .05) restored to normal levels by the positive control, tamoxifen. In terms of histological investigation, the administration of hypophyllanthin to rats with tumours revealed a moderate reduction in the amount of necrotic cells (NCs). In comparison to untreated cancer-bearing rats, cancer-bearing rats treated with hypophyllanthin demonstrated tumours of a smaller size. This prior study equally suggested that the potent mammary tumour inhibition of hypophyllanthin might have limited the rate of necrosis. The findings suggest that the *in vitro* and *in vivo* anticancer activities of hypophyllanthin might be mediated by a variety of mechanisms. The probable mechanism that might contribute to the anticancer activity of hypophyllanthin is the direct free-radical scavenging activity, followed by the inhibition of cytochrome P450 enzymes and blockade of the estradiol biosynthesis by inhibiting aromatase and steroid dehydrogenase enzymes (Parvathaneni et al., 2014a). Taken together, it is worth to further explore the anticancer activity of hypophyllanthin on different signaling pathways mediating antiproliferative effects on cancer cells while establishing the safety profile of the compound as a potential new therapy for a specific type of cancer.

### 4.4 Hepatoprotective activity

Hypophyllanthin, which is prevalent in *Phyllanthus* species, is characterised by its ability to protect the liver against toxic substances. One of the earliest studies examining the hepatoprotective potential of hypophyllanthin found that the hexane extract of *P. niruri* demonstrated efficacy against both carbon tetrachloride- and galactosamine-induced cytotoxicity in primary cultured rat hepatocytes (Syamasundar et al., 1985). Although the hexane extract contained phyllanthin, hypophyllanthin, triacontanal, and triacontanol, only phyllanthin, and hypophyllanthin exhibited hepatoprotective activity against carbon tetrachloride- and galactosamine-induced cytotoxicity. The mean glutamic-pyruvic transaminase (GPT) activity of carbon tetrachloride (82%) and galactosamine (86%) was lower in the 1.0 mg/mL hypophyllanthin group than in the control group (100%). The researchers also observed that phyllanthin and hypophyllanthin significantly inhibited carbon tetrachloride-induced cell damage. The findings highlighted that the overall hepatoprotective effect of *P. niruri* was due to the synergistic effect of hypophyllanthin and phyllanthin.

Subramanian et al. (2019) performed molecular docking study to explore the potential action of hypophyllanthin, phyllanthin, and gallic acid in blocking the active sites of the liver alcohol dehydrogenase enzyme which is involved in the oxidation of alcohols including methanol and ethanol to acetaldehyde, a highly toxic substance and known carcinogen. The results depicted that the binding efficiency of the compounds, phyllanthin (−2.37 kcal/mol), hypophyllanthin (−3.23 kcal/mol) and gallic acid (−5.85 kcal/mol), arranged in the order of increasing binding efficiency, was comparable to that of the standard, 4-methyl pyrazole (−4.18 kcal/mol).

In contrast, despite several previous studies reporting the link between hypophyllanthin and hepatoprotective activity, it is still debatable if hypophyllanthin can act as a hepatoprotective agent. As a result, some researchers have hypothesised that the hepatoprotective activity exhibited by *Phyllanthus* species is unrelated to the presence of hypophyllanthin. *Phyllanthus* species was still able to display hepatoprotective effects despite the absence of hypophyllanthin and phyllanthin. In a study conducted by Srirama et al. (2012), the methanol extracts of *P. polyphyllus, P. emblica,* and *P. indofischeri* which lacked hypophyllanthin exhibited a very high level of hepatoprotective activity at comparatively low EC_50_ values of 12, 19, and 28 μg/mL, respectively, as compared to the positive control, silymarin, which showed an EC_50_ of 32 μg/mL. Both aqueous and methanolic extracts of *P. virgatus* also demonstrated positive hepatoprotective activity. Non-etheless, Srirama et al. (2012) found that the methanol extract of *P. amarus* contained 2.3% of phyllanthin and 1.1% hypophyllanthin but no hepatoprotective activity was recorded at a concentration of 50 μg/mL. This study suggested that the hepatoprotective activity attributed to the investigated species (*P. virgatus, P. polyphyllus, P. emblica,* and *P. indofischeri*) might be due to other factors and active principles. It was also reported in another study that *P. polyphyllus* exhibited hepatoprotective and antioxidant activities in acetaminophen-induced hepatotoxicity rat subjects despite the absence of hypophyllanthin in this plant (Rajkapoor et al., 2008). Hence, it was suggested that the hepatoprotective activity of *P. polyphyllus* was attributed to the inhibition of lipid peroxidation, enhancement of the antioxidant enzyme levels, and free radical scavenging. The researchers also posited that the flavonoids present in this plant could be the active principle responsible for the hepatoprotective activity. Based on the above findings, the hepatoprotective effect of the extracts of *Phyllanthus* species may be contributed by the synergistic effects of all their bioactive chemical constituents.

Hypophyllanthin has been demonstrated to be a powerful, mechanism-based inhibitor of cytochrome P3A4 (CYP3A4), one of the subtypes of cytochrome P450 (CYP450) in the liver despite its hepatoprotective activity remains unclear (Taesotikul et al., 2011). Since most medications are processed in CYP450, this bioactivity will lead to an enhanced risk for herb-drug interactions. The inhibitory effects of *P. amarus* and its major phytochemicals phyllanthin and hypophyllanthin on CYP isoforms were determined using human liver microsomes and selective substrates (Taesotikul et al., 2011). The results demonstrated both ethanol and aqueous extracts of *P. amarus* dose-dependently inhibited P450 subtypes, CYP1A2, CYP2D6, CYP2E1, and CYP3A4. The study demonstrated that hypophyllanthin exhibited a significant inhibitory effect on CYP3A4 activity with an IC_50_ value of 2.90 ± .68 μM. In addition, phyllanthin and hypophyllanthin were potent mechanism-based inhibitors of CYP3A4 with K I values of 1.75 ± 1.20 μM and 2.24 ± 1.84 μM and k inact values of .18 ± .05/min and .15 ± .06/min, respectively. Besides, the K inact/K1 ratios of hypophyllanthin (83.21 ± 29.26/min/nM) reported in this study were higher than those reported for some therapeutic drugs that act as mechanism-based inhibitors of CYP3A4. These results suggested that co-administration of *P. amarus* with drugs that are metabolized by CYP3A4 may potentially result in herb-drug interactions. The findings reflected that the inhibition of hypophyllanthin against CYP3A4 was time-dependent, which suggests that hypophyllanthin may be a mechanism-based inhibitor of CYP3A4. Additionally, hypophyllanthin has a promising future for hepatitis therapy, particularly for the treatment of the hepatitis C virus.

The ethanol extract of *P. niruri* has been evaluated for its anti-hepatitis C virus (HCV) activity in *vitro* assay in immortal (Huh 7it) cells (Wahyuni et al., 2019). HCV is one of the causes or factors associated with the incidence of chronic liver disease, especially hepatitis and hepatocellular carcinoma. The extract inhibited HCV with an IC_50_ value of 4.14 μg/mL, without any potential development of toxicity in the host cell. The extract also exhibited stronger HCV inhibition in the entry step (70%) relative to the post-entry step (50%). The combination of the extract with simeprevir (NS3 protease inhibitor) could also boost the antiviral activity of the latter with an IC_50_ value of 3.54 nM. The increase in the activity of simeprevir was up to 4-fold compared to its single treatment. This outcome was further supported by molecular docking analysis to predict the interaction of phyllanthin and hypophyllantin, known compounds of *P. niruri* against HCV receptor. The results depicted that both lignans exhibited strong interaction and bound to 4-glycosaminoglycans (4-GAG) receptor that was crucial for HCV entry to the cells of hepatocytes. These results suggested that the lignans of ethanol extract of *P. niruri* may be good candidates for the development of anti-HCV drugs. Although its potential as a hepatoprotective agent is debatable, it has been demonstrated that hypophyllanthin affected the liver by inhibiting CYP3A4. As a result, detailed mechanistic insights focusing on its molecular pathways should be studied further to better understand its mechanism of action.

### 4.5 Anti-hyperuricaemic activity

Hyperuricaemia is a condition characterised by a high amount of uric acid in the blood, either resulting from abnormally excessive uric acid production or low uric acid removal from the body. It is a metabolic anomaly associated with chronic diseases, such as gout, hypertension, and renal failure (Ghei et al., 2002). The *in vivo* antihyperglycaemic effect of the methanol extract of *P. niruri* have been reported by Murugaiyah and Chan (2006) in potassium oxonate- and uric acid-induced hyperuricemic male Sprague-Dawley rats. A remarkable improvement was observed after 7 days of treatment in which the plasma uric acid level was decreased by 57.08%, 59.44%, 77.47%, and 83.91% with doses equivalent to 100, 200, 500, and 1,000 mg/kg, respectively (*p* < .001). Hence, the efficacy of the methanol extract treatment was strongly dependent on the administered dose. Besides, the positive controls, allopurinol at 50 mg/kg, benzbromarone at 50 mg/kg, and probenecid at 200 mg/kg demonstrated a similar trend in the reduction of plasma uric acid levels (82.19%, 87.77%, and 69.10%, respectively) at the same treatment duration. Overall, the activity of positive controls was slightly stronger than the methanol extracts. The extract was fractionated by resin chromatography and the active fraction was subjected to bioassay-guided isolation to afford the lignans, phyllanthin, hypophyllanthin, and phyltetralin. The lignans could also reduce the plasma uric acid level of the hyperuricemic rats in a dose-dependent manner. Phyllanthin showed the strongest effect on the reduction of plasma uric acid level of hyperuricemic rats to its normal level, comparable to that of positive controls, allopurinol, benzbromarone and probenecid which are used clinically for the treatment of hyperuricemia and gout. At a relatively low dose of 10 mg/kg, hypophyllanthin aided in lowering plasma uric acid level by 64.47% (*p* < .01). Hypophyllanthin recorded a significant higher uric acid clearance (.19 ± .05 mL/(kg/hr)) as compared to the normal (.13 ± .04 mL/(kg/hr)) and hyperuricemic subjects (.03 ± .04 mL/(kg/hr)). Anti-hyperuricaemic effect of the lignan constituents of *P. niruri* including hypophyllanthin was comparable to that of the clinically used drugs (allopurinol, benzbromarone, and probenecid). Thus, hypophyllanthin has high potential to be used for the treatment of gout and hyperuricemia-related diseases. This effect is likely *via* its uricosuric action, leading to xanthine oxidase inhibition (Murugaiyah and Chan 2009).

### 4.6 Vasoactive activity

The lignans, hypophyllanthin, phyllanthin, and nirtetralin, isolated from *P. niruri* were found to have a strong vasodilating effect on the arterial tissue and prevented the endothelin (ET-1) from attaching to its receptor, the endothelin (ET-1) type A receptor of rat thoracic aortic smooth muscle cells (Hussain et al., 1995). The lignans were also found to inhibit ET-1 binding to recombinant human ET-1 type A receptor expressed in Chinese hamster ovary cells. However, they were inactive against the ET-1 type B receptor, thereby establishing them as selective inhibitors that could prevent the formation of the complex between ET-1 and ET-1 type A receptor. Among the lignans, hypophyllantin was the most potent with an IC_50_ value of 40 µM which was significantly lower than that of phyllanthin. Hypophyllanthin was also found to inhibit ET-1-induced elevated acidification rate that was consistent with ET-1 antagonistic activity.

Inchoo et al. (2011) investigated the modulating effects of hypophyllanthin and phyllanthin on vascular tension, using an *in vitro* model of isolated rat aorta. The prolonged constriction brought on by phenylephrine (PE) was considerably and dose-dependently relaxed by both lignans. The researchers also exhibited that the effect of the lignans on vasorelaxation responses of the aortic rings was not significantly altered by the absence of intact endothelium. Endothelium cells are essential due to their ability in secreting vasodilators, such as nitric oxide (NO), prostacyclin, and endothelium-derived hyperpolarising factor. Despite its ability to regulate vasoconstriction, the lignans were unable to stimulate the release of these endothelium-derived relaxing factors (EDRF). The compounds could inhibit aortic muscle contraction induced by PE (1 μM) or KCl (40 mM) as well as the spontaneous contraction of the Ca^2+^-depleted muscle. At 100 μM, both lignans attenuated PE-mediated contraction in Ca^2+^-free condition but failed to inhibit caffeine-induced contraction. In the presence of PE, the control (DMSO) recorded the highest percentage of contraction (90%), followed by hypophyllanthin (60%) and phyllanthin (20%). Taken together, phyllanthin and hypophyllanthin could modulate the vascular tension *via* the endothelium-independent mechanisms. The results suggested that the modulation of the vascular tension effects by hypophyllanthin and phyllanthin could possibly involve the blockade of Ca^2+^ entry into vascular smooth muscle cells and PE-induced Ca^2+^ released from sarcoplasmic reticulum.

The positive effect of phyllanthin and hypophyllanthin as vasodilators demonstrated in these studies can be further explored in order to highlight their potential role to treat hypertension. In hypertension there is endothelial dysfunction of the vascular smooth muscle cells, resulting in loss of normal vasorelaxant function and inability of arteries to dilate appropriately in response to increased blood flow in either a systemic or regional vascular bed.

### 4.7 Estrogenic activity

Islam et al. (2008a) investigated the protective effect of hypophyllanthin and phyllanthin isolated from *P. amarus* on disrupted estrous cycle and follicular growth in virgin Wister rats treated with a carbamate insecticide, carbofuran. Carbofuran is a cholinesterase enzyme inhibitor that could have altered the normal mechanism of hormonal secretion and was responsible for the disruption in the reproductive cycle. Both lignans have been shown to be capable of preventing the lethal effect of carbofuran by regulating the hormonal secretion. As phytoestrogens, the lignans were capable of interacting with estrogen receptors and produced estrogen-type activity either as agonists or antagonists. Hypophyllanthin and phyllanthin could be transformed into beneficial enterolignans upon metabolism in the intestines. Enterolignan is a macromolecule that could increase and repair abnormal oestrous cycle in rats exposed to toxic substances. The authors hypothesized that a mixture of phyllanthin and hypophyllanthin in a ratio of 1:1 would have a synergistic effect in restoring the oestrous cycle. However, it was not being pointed out in this study whether the observed toxicity was due to the direct effects on the ovary or indirectly through action on the hypothalamus and/or pituitary, or by desensitizing the ovary to gonadotropins. Hence, additional research is required to support the bioactivity of the lignans as phytoestrogens.

### 4.8 Anti-allergic activity

Allergy is one of the symptoms of improper control of the immune system. An allergic reaction is generated by mast cell degranulation in response to allergen exposure. Primary treatments for immunoglobulin E (IgE)-mediated allergy disorders include antiIgE serum, antihistamines, and mast cell stabilisers. Abd Rani et al. (2021) investigated the antiallegic effect of the 80% ethanol extract of *P. amarus* and its compounds by determining the concentration of allergy markers release from rat basophilic leukemia (RBL-2H3) cells. The release of both biomarkers; beta-hexosaminidase and histamine, were not inhibited by the extract thus allowing the mast cell degranulation. However, mild antihistamine activity of the extract was observed. Among the compounds, only the lignans, phyllanthin, hypophyllanthin, and niranthin inhibited the release of β-hexosaminidase. The antihistamine activity was evaluated by conducting a competition radioligand binding assay on the histamine 1 receptor (H1R). Hypophyllanthin displayed the highest affinity for the radioligand competition binding assay (Ki value: .04 nM), followed by niranthin (Ki value: .44 nM), and phyllanthin (Ki value: 129.1 nM). Hypophyllanthin also demonstrated greater activity (a Ki value of .04 nM) compared to chlorpheniramine (Ki value = .1447 nM), suggesting that hypophyllanthin might have a promising therapeutic property as an antihistamine. Through a molecular docking study, hypophyllanthin was identified to exhibit favourable binding in the H1R binding site. Thus, potential antiallergic activity of hypophyllanthin maybe be due to the inhibition of the activation of the H1 receptor. Based on the *in vitro* studies, hypophyllantin was shown to possess anti-allergic properties by inhibiting H1 receptor activation. Consequently, we can further investigate the anti-allergic effects of hypophyllantin by using animal models treated with the bioactive compound to determine its systemic role.

## 5 Pharmacokinetic and bioavailability studies of hypophyllanthin

A method developed by Murugaiyah and Chan (2007b) was used to conduct a pharmacokinetic study of lignans including hypophyllanthin from *P. niruri* in rats. In this work, hypophyllanthin and the other lignans could be clearly recognised and detected in the rats’ plasma even after 10 h of oral lignan treatment. The lignans (phyllanthin, hypophyllanthin, phyltetralin and niranthin) administered intravenously were slowly eliminated from the body with a mean clearance of .04, .01, .03, and .02 L/kg h and a mean half-life of 3.56, 3.87, 3.35, and 4.40 h. After 1 h administration their peak plasma concentration was .18, .56, .12, and .62 μg/ml, respectively. However, their absorption was incomplete with a calculated absolute oral bioavailability of .62%, 1.52%, 4.01%, and 2.66% for phyllanthin, hypophyllanthin, phyltetralin, and niranthin, respectively. Due to its high lipophilicity and low water solubility, hypophyllanthin is a very poor candidate for oral administration. Hypophyllanthin demonstrated peak plasma concentrations up to .15–.22 g mL^−1^ within 1 h after oral administration, followed by a gradual decline to 0 after 24 h. However, due to its low aqueous solubility, oral absorption of hypophyllanthin was incomplete and had poor under plasma concentration (AUC) (Murugaiyah and Chan, 2007b).

A validated RP-HPLC–PDA method was developed to determine the concentrations of hypophyllanthin and phyllanthin in various plasma samples of Sprague Dawley rats in an oral pharmacokinetic study. There was a rapid rise with a Tmax of 1 h followed by a gradual decline to 0 after 24 h for both lignans. A non-linear increase in AUC0→24 h values with increase in their oral doses was exhibited by both compounds. Since the lignans were lipophilic, they could penetrate the gastrointestinal tract. The C_max_ (ng/mL) values for administered three oral doses (2.5, 5, and 10 mg/kg) of hypophyllanthin were .68 ± .76, 1.35 ± .23, and 2.45 ± .33, respectively. The developed method could be used successfully in the pharmacokinetic study of lignans in rats as it could measure the concentrations of lignans in rat plasma up to 12 h after oral administration (Parvathaneni et al., 2014b).

A rapid and sensitive HPLC–MS/MS method was developed and validated to simultaneously determine the concentrations of hypophyllanthin together with nirtetralin, phyllanthin and niranthin from *P. urinaria* in rat plasma in a pharmacokinetics study. The spectra showed the presence of sodium adduct [M + Na]^+^ ions as molecular ions for all lignans when utilising electrospray ionisation (ESI) positive ionisation mode. Hypophyllanthin did not produce protonated adduct [M + H]^+^ ions while the ions were produced at a low level for phyllanthin (Fan et al., 2015). In another study, metabolite changes of aqueous ethanol extracts of *P. niruri* were determined by using a proton nuclear magnetic resonance (1H-NMR)-based metabolomics approach. Freeze-dried sample of *P. niruri* extracted with 80% ethanol was found to contain the highest amounts of hypophyllanthin and phenolic compounds based on the loading plot of principal component analysis (PCA). The distinctive binned signals of hypophyllanthin were recorded at chemical shift 5.62 ppm based on ^1^H-NMR signals. The partial least-square (PLS) results showed that the phytochemicals, including hypophyllanthin were correlated with antioxidant and α-glucosidase inhibitory activities of the extracts, which were higher in the sample material extracted with 80% ethanol (Mediani et al., 2017).

## 6 Toxicology

High interest in the medical benefits of hypophyllanthin has led to further investigations of its pharmacological and toxicological properties. Numerous *in vitro* studies have reflected that hypophyllanthin has high cell viability in immune cells and was less likely to induce toxic effects on the host cells. In a cell viability assay using the standard trypan blue exclusion method in polymorphonuclear leukocyte (PMNs), the highest concentration in which the cells were viable (>95%) after 2 h incubation with hypophyllanthin was 50 μg/mL (Yuandani et al., 2013). Jantan et al. (2014) reported that the cell viability of hypophyllanthin in PMNs and monocytes were greater than 95% at a safe concentration range from .3125 to 5 g/mL. While measuring the concentration of allergy markers released from rat basophilic leukaemia (RBL-2H3) cells, Abd Rani et al. (2021) also found that ketotifen fumarate (positive control) and hypophyllanthin concentrations below 25 g/mL were safe in the cell and maintained approximately 70% viability for antiallergic activity. Conclusively, hypophyllanthin has a high potential to modulate the cellular immune response without inducing detrimental and damaging toxic effects on the host cells. Referring to the high cell viability, the extracts and compounds were not toxic to immune cells and may potentially alter the cellular immune response in reaction mixture, including allergic reactions.

Kushwaha et al. (2013) conducted an *in vivo* study in female albino rats to evaluate the acute toxicity of standardised methanol extract of *P. amarus* which contained phyllanthin (8.91% w/w) and hypophyllanthin (5.01% w/w). The extract was administered orally to the treated groups in doses of 300, 600, 2000, and 5,000 mg/kg body weight, while the control group received a standard laboratory diet and water *ad libitum* in accordance with the Organisation for Economic Cooperation and Development (OECD) guideline 423 with some modifications. No mortality or significant changes of the general behaviour, body weight, gross appearance of internal organs observed over 14 days. Likewise, histological profile of the liver indicated the non-toxic nature of phyllanthin and hypophyllanthin in the extract. Biochemical studies also depicted no significant changes in the levels of alanine aminotransferase (ALT), aspartate aminotransferase (AST), albumin, triglycerides, cholesterol and albumin. Besides, no evidence of congestion or haemorrhage in the sinusoids and hepatocytes and no fatty changes, centrilobular necrosis, and changes in the number of Kupffer cells in the liver. The blood pressure was normal and no evidence of nephrotoxicity and acute severe hepatotoxicity in the kidney and liver, respectively. Therefore, the acute toxicity tests indicated that the methanol extract of *P. amarus* containing phyllanthin and hypophyllanthin was non-toxic and safe as evidenced by its high LD_50_ > 5,000 mg/kg body weight of the rats.

## 7 Conclusion

Hypophyllanthin has been investigated for a diversity of beneficial pharmacological effects such as immunomodulation, anti-inflammatory, anticancer, hepatoprotective, anti-hyperuricemic, vasoactive, and anti-allergic activities. The first two pharmacological activities, i.e., immunomodulation and anti-inflammatory activities have been the major interest of many researchers. The immunomodulating activity of hypophyllanthin has been demonstrated in many *in vitro* and *in vivo* experiments. In several *in vitro* studies, the immunosuppressive effects of hypophyllanthin on the immune responses by targeting inflammation-associated signaling pathways have been demonstrated. There are several studies on the *in vivo* immunomodulatory effects of *Phyllanthus* species where hypophyllanthin was identified as one of the major contributor to the suppressive effects on the immune responses. However, *in vivo* immunomodulatory effects of hypophyllanthin on cellular immune responses in animal model have not been reported. Hypophyllanthin has been shown to exhibit anti-inflammatory activity in many *in vitro* and *in vivo* experiments. Many *in vivo* studies on the anti-inflammatory activities of *Phyllanthus* species in various animal models were associated with the anti-inflammatory properties of hypophyllanthin. Hypophyllanthin demonstrated moderate anti-tumour activity that was typically concentration dependent. Hypophyllanthin has been shown to possess phytoestrogen effect that assists in restoring the oestrous cycle. Although its potential as a hepatoprotective agent is debatable, it has been demonstrated that hypophyllanthin affected the liver *via* inhibiting CYP3A4. It could also treat and regulate excessive plasma uric acid levels and modulate vascular tension either with or without the presence of endothelial cells that act to release vasodilators. Hypophyllanthin also possess antiallergic activities *via* inhibiting the activation of the H_1_ receptor. Furthermore, the presence of other phytochemicals such as phyllanthin, has enhanced certain bioactivities of hypophyllanthin, possibly due to their synergistic effects. In conclusion, hypophyllanthin shows promise as a lead compound for the discovery of drug candidates, especially for the development of therapies for inflammatory and immune-related diseases.

## 8 Future Perspectives

Further studies on the pharmacological activities and their mechanisms of action need to be performed before hypophyllanthin can be used as a lead molecule for further development into a drug candidate, especially for development of therapies for inflammatory and immune related diseases. The compound has to be further assessed for its efficacy and safety by investigating its bioavailability, pharmacokinetics and pharmacodynamic properties in various disease animal models as well as toxicity studies, before submission to clinical studies. A few animal studies have demonstrated promising immunomodulating, anti-inflammatory, anticancer, hepatoprotective, and anti-hyperuricaemic activities. However, no human clinical studies have been conducted to evaluate the therapeutic potential of hypophyllanthin in immunomodulation, inflammation, anticancer, hepatoprotective, anti-hyperuricemic, vasoactive, or anti-allergic activities. Therefore, additional research including randomised human clinical studies should be conducted in order to establish an evidence-based clinical profile and to evaluate the therapeutic potential and safety of hypophyllanthin in the treatment of the above-mentioned diseases.

## 9 Limitation

Despite the fact that hypophyllanthin has the potential to be developed as an anti-inflammatory and immune-drug agent, there are various challenges and limitations that may impede its development in the meantime. One of the difficulties is that the natural source of the compound is consistently unreliable. The biosynthesis of bioactive chemicals such as hypophyllanthin may be heavily influenced by genetic factor, geographical considerations as well as environmental conditions. For example, it was discovered that several *Phyllanthus* species, such as *P. amarus*, produced much more hypophyllanthin when grown in heavy metal-rich soils. As a result, obtaining constant quality and quantity of hypophyllanthin in varied settings is a difficult issue for researchers.
